# New-born females show higher stress- and genotype-independent methylation of *SLC6A4* than males

**DOI:** 10.1186/s40479-015-0029-6

**Published:** 2015-04-15

**Authors:** Helene Dukal, Josef Frank, Maren Lang, Jens Treutlein, Maria Gilles, Isabell AC Wolf, Bertram Krumm, Renaud Massart, Moshe Szyf, Manfred Laucht, Michael Deuschle, Marcella Rietschel, Stephanie H Witt

**Affiliations:** Department of Genetic Epidemiology in Psychiatry, Central Institute of Mental Health, Medical Faculty Mannheim, University of Heidelberg, Mannheim, Germany; Department of Psychiatry and Psychotherapy, Central Institute of Mental Health, Medical Faculty Mannheim, University of Heidelberg, Mannheim, Germany; Department of Biostatistics, Central Institute of Mental Health, Medical Faculty Mannheim, University of Heidelberg, Mannheim, Germany; Department of Pharmacology and Therapeutics, McGill University, Montreal, QC Canada; Department of Child and Adolescent Psychiatry and Psychotherapy, Central Institute of Mental Health, Medical Faculty Mannheim, University of Heidelberg, Mannheim, Germany

**Keywords:** Serotonin transporter, *SLC6A4*, 5-HTTLPR, Methylation, Early life stress, Sex

## Abstract

**Background:**

Research has demonstrated an association between exposure to early life stress and an increased risk of psychiatric disorders in later life, in particular depression. However, the mechanism through which early life stress contributes to disease development remains unclear. Previous studies have reported an association between early life stress and altered methylation of the serotonin transporter gene (*SLC6A4*), a key candidate gene for several psychiatric disorders. These differences in methylation are influenced by sex and genetic variation in the *SLC6A4-*linked polymorphic region (5-HTTLPR). Furthermore, one study indicated that stress during pregnancy may induce methylation changes in *SLC6A4* in the newborn. The present study is the first to investigate whether early life stress during pregnancy impacts on *SLC6A4* methylation in newborns, taking into account the influence of genetic variation and sex.

**Methods:**

Cord blood was obtained from newborns with high (n = 45) or low (n = 45) early life stress, defined as maternal stress during pregnancy. The effect on methylation of early life stress, 5-HTTLPR genotype, and sex was assessed at four cytosin-phosphate-guanine dinucleotide (CpG) sites in the promoter associated CpG island north shore (CpG 1 to 4). The epigenetic analyses focused on these CpG sites, since research has shown that CpG island shore methylation has functional consequences.

**Results:**

Significant sex-specific methylation was observed, with females displaying higher methylation levels than males (p < 0.001). Importantly, this effect was influenced by neither early life stress nor genotype.

**Conclusions:**

The present data suggest that sex-specific methylation of *SLC6A4* is present at birth, and is independent of early life stress and 5-HTTLPR genotype. This may contribute to the sex-specific prevalence of depression.

**Electronic supplementary material:**

The online version of this article (doi:10.1186/s40479-015-0029-6) contains supplementary material, which is available to authorized users.

## Background

The serotonin transporter gene (*SLC6A4*) is one of the most extensively investigated genes in psychiatry, and has been implicated in a wide range of psychiatric disorders [1]. Research has shown that variation in this gene is associated with stress-related psychiatric disorders, such as major depression and anxiety [2]. The most consistently reported association is with variation in the *SLC6A4-*linked polymorphic region (5-HTTLPR). This 5-HTTLPR consists of a 44-base-pair insertion/deletion polymorphism in the 5′ regulatory promoter region of *SLC6A4*, which results in a short (S) and long (L) allele of the gene and a single nucleotide polymorphism in the L allele of 5-HTTLPR (rs25531 A/G) which leads to a triallelic locus (S/LA/LG) [3]. The S allele has been reported to reduce transcriptional activity of the gene promoter *in vitro,* and to be associated with depression-related personality traits [4,5]. Moreover, research has identified a gene x environment interaction for life stress and the 5-HTTLPR genotype in adults with depression [6]. In comparison to L homozygotes, S allele carriers showed higher levels of depressive symptoms, diagnosable depression, and suicidality.

Furthermore, numerous studies have demonstrated methylation differences in *SLC6A4* in individuals with depressive symptoms [7], depression [8], and a history of early life stress [9-14]. Sex- [8,9] and genotype-effects have also been described [10-12].

Several studies have demonstrated that early life stress has a major impact on mental health in later life. Childhood adversity is associated with increased vulnerability to several psychiatric disorders, in particular mood, anxiety, and personality disorders [15]. Furthermore, maternal stress during pregnancy has been associated with distinct behavioral disturbances in the offspring in later life [16]. In particular, maternal stress during pregnancy appears to have major effects on the stress response and the propensity to depression in the offspring [17]. To date, only one study has analyzed the influence of depressed maternal mood during pregnancy on serotonin transporter methylation levels in the newborn. In this study, increased 2^nd^ trimester depressed maternal mood was associated with decreased maternal and infant serotonin transporter promoter methylation [18]. However, the authors did not take into account the possible effect of variation in 5-HTTLPR genotype and sex.

A previous study has shown that methylation of *SLC6A4* CpG island shore CpG sites has functional consequences [19]. Generally, methylation of DNA cytosines in the context of cytosin-phosphate-guanine (CpG) dinucleotides is a covalent modification of DNA with a methyl group. The latter is introduced into the DNA by an enzymatic reaction catalyzed by DNA methyltransferases [20]. CpG dinucleotides are often clustered in CpG islands at the promoter region of genes. In general, methylation leads to gene silencing via reduced trancriptional activity [21]. Previous authors have hypothesized that early life adversity results in DNA methylation and life-long phenotypes [20].

Therefore, the aims of the present study were to determine 1) whether exposure to maternal stress during pregnancy leads to higher methylation levels in *SLC6A4* in newborns; and 2) whether this is influenced by sex and 5-HTTLPR genotype. The analyses focused on four previously reported functional serotonin transporter gene CpG island north shore CpG sites [19].

## Methods

### Study participants

Data were obtained from a cohort of mothers and their infants (n=410), respectively recruited during the third trimester of pregnancy (4–8 weeks prior to term) from the Rhine-Neckar Region of Germany. The study protocol was approved by the Ethics Committee of the Medical Faculty Mannheim of the University of Heidelberg. The study was conducted in accordance with the Declaration of Helsinki. All mothers provided written informed consent prior to participation. A structured interview and questionnaires were used for risk factor assessment. Inclusion criteria for mothers were: main caregiver; German-speaking; and age 16–40 years. Exclusion criteria for mothers were: hepatitis B, hepatitis C, or human immunodeficiency virus (HIV) infection; any current psychiatric disorder requiring inpatient treatment; any history or current diagnosis of schizophrenia/psychotic disorder; or any substance dependency other than nicotine during pregnancy. Exclusion criteria for infants were: birth weight < 1,500 gram; gestational age < 32 weeks; or the presence of any congenital diseases, malformations, deformations, and/or chromosomal abnormalities.

A broad range of environmental and sociodemo-graphic risk factors, prenatal medical risk factors, general medical characteristics, and psychosocial risk factors were assessed (Table 1). Eight main stressor variables were derived from eight different questionnaires (Perceived stress scale [PSS], Prenatal distress questionnaire [PDQ], Life experiences survey [LES], Social support questionnaire [SOZ_U], Mini-International Neuropsychiatric Interview [M.I.N.I.], Edinburgh postnatal depression scale [EPDS], State-trait anxiety inventory [STAI-S & STAI-T], Anxiety screening questionnaire [ASQ]) in order to represent a variety of prenatal adversities and to take the following three dimensions of stress into account: a) maternal psychopathology (primarily depressive and anxious symptoms); b) perceived stress; and c) socioeconomic and psychosocial stress. All data were assessed at two timepoints: in the 3^rd^ trimester and at birth (see Table 1 and Additional file 1 for details). In addition, an “adversity score” was calculated. This was obtained by summing up all positive responses to the following 12 stressful dichotomous (yes/no) stressful adverse prenatal conditions and environmental circumstances: 1) current and lifetime M.I.N.I. (Mini International Neuropsychiatric Interview) diagnosis; 2) current psychiatric diagnosis; 3) not cohabiting with partner; 4) low levels of encouragement from the partner; 5) separation(s) in the last year; 6) daily verbal conflict; 7) physical conflict; 8) crowded living conditions (≤ one room/person); 9) no academic qualification; 10) no professional education; 11) low net family income; and 12) debt (see Table 2 for details). Further information on demographic variables is provided in (Additional file 2: Table 1).

**Table 1:** **Phenotypic assessment of mothers and infants at two time points**

	**Prenatal/3** ^**rd**^ **trimester**	**Perinatal/birth**
***Exposure to ELS***	Perceived stress (PSS) [33]	Pre- and perinatal complications
	Prenatal distress (PDQ) [34]	Perinatal stressors (e.g., asphyxia, cesarian, preterm birth)
	Life events (LES) [35]	Pregnancy & obstetric history (birth weight, gestational age, birth complications)
	Social support (Soz-U.) [36]	
	Socio-demographic data	
	Maternal health risk behavior (e.g. smoking)	
	Psychosocial risks	
***Maternal mental & physical health***	Maternity log-book data	
	Semi-standardized neuropsychiatric diagnostic interview (MINI) [37]	
	Depression screening (EPDS) [38]	
	Anxiety screening (STAI-S [39], STAI-T [39], ASQ [40])	
	Anthropometry	
	Individual & family history of metabolic and other medical disorders	

ELS = early life stress; PSS = perceived stress scale; PDQ = prenatal distress questionnaire; LES = life experiences survey; Soz-U = social support questionnaire; M.I.N.I. = Mini-international neuropsychiatric interview; EPDS = Edinburgh postnatal depression scale; STAI-S & STAI-T = state-trait anxiety inventory; ASQ = anxiety screening questionnaire.

**Table 2:** **Psychopathology and socioeconomic-, psychosocial-, and perceived stress of the extreme group mothers (all data: mean ± SD or percentage)**

**Variable**	**High prenatal ELS (n = 45)**	**Low prenatal ELS (n = 45)**	**p value**
*Maternal psychopathology*			
EPDS Score^1^	12.33 ± 4.70	1.27 ± 1.45	0.000
STAI-S Score^1^	51.72 ± 8.05	27.41 ± 3.87	0.000
STAI-T Score^1^	47.93 ± 8.41	28.04 ± 3.58	0.000
ASQ Score^1^	4.11 ± 2.14	0.53 ± 0.72	0.000
M.I.N.I. Diagnosis^2^			
None	51.1%	91.1%	0.004
Depressive disorder	40%	6.7%	0.015
Anxiety disorder	8.9%	2.2%	ns
Current psychiatric disorder^2^			
None	62.2%	97.8%	0.005
Depressive disorder	26.7%	0%	0.015
Anxiety disorder	11%	2.2%	ns
*Perceived stress*			
PSS Score^1^	30.84 ± 5.64	12.84 ± 4.1	0.000
PDQ Score^1^	19.95 ± 7.56	5.95 ± 2.95	0.000
*Socioeconomic and psychosocial stress*			
LES-negative events Score^1^	6.75 ± 4.39	1.80 ± 1.29	0.010
Soz-U Score^1^	39.58 ± 3.85	51.89 ± 4.32	0.000
Living without a partner^2^	20%	0%	0.004
Encouragement (Partner)^2^	73.3%	100%	ns
Separation(s) in the last year^2^	24.4%	2.2%	0.002
Daily arguments^2^	11.1%	2.2%	ns
Physical conflicts within the preceding 12 months^2^	33.3%	2.2%	0.003
Composition of household > one person/room^2^	40.5%	0%	0.001
No academic qualification^2^	4.4%	2.2%	0.050
No professional education^2^	17.7%	2.2%	0.043
Monthly income per household ≤ 1,750 Euro^2^	42.2%	0%	0.001
Debt^2^	24.4%	2.2%	0.002

SD = standard deviation; ns = not significant. ^1^The first eight main variables of the principal component analysis (PCA), ^2^The twelve prenatal stressors used to generate the adversity score as the ninth main variable of the PCA.

To obtain a homogeneous composite measure of prenatal stress, a principal component analysis (PCA) was performed using the eight main stressor variables and the total adversity score. This yielded a first principal component (PC1) that explained around 60% of the common variance. PC1 was then used to determine two extreme groups: 45 infants (24 females; 21 males) with extremely high levels of prenatal early life stress; and 45 infants (27 females; 18 males) with extremely low levels of prenatal early life stress [22].

Demographic characteristics included in the methylation analysis were maternal age (maternal age high prenatal stress, 29.60 ± 5.61; maternal age low prenatal stress, 32.73 ± 3.69) and the sex of the newborn (high prenatal stress female 53.3%; low prenatal stress female 60%).

### 5-HTTLPR rs25531 multimarker genotyping

Simultaneous genotyping of 5-HTTLPR and rs25531 was carried out by polymerase chain reaction (PCR) according to Wendland et al. [23]. In a total volume of 20 μl, 60 ng of genomic DNA was amplified in the presence of 1 x Master Mix (Promega) using the following primers: forward primer: 5′-TCCTCCGCTTTGGCGCCTCTTCC-3′; reverse primer: 5′-TGGGGGTTGCAGGGGAGATCCTG-3′. The PCR conditions were: 5 minutes at 95°C, followed by 35 cycles of 30 seconds at 95°C, 90 seconds at 70°C, 60 seconds at 72°C, and a final extension of 10 minutes at 72°C. Subsequently, complete PCR product was digested with HpaII (New Enlgand Biolabs) in a 25 μl reaction assay containing 1xNEBuffer 1 at 37°C for 4 h. Then, 15 μl of the digested PCR products were run on a 2.5% agarose gel and visualized with ethidium bromide staining. For the 5-HTTLRP L allele, a band of 512 basepairs (rs25531, A allele, uncut) or 402 basepairs and 110 basepairs (rs25531, G allele, cut) was visible. For the 5-HTTLRP S allele, a band of 469 basepairs was visible. The 5-HTTLPR rs25531 multimarker genotype is combined according to its *SLC6A4* mRNA expression into high expressing genotype 5-HTTLPR LA and low expressing genotype 5-HTTLPR S and LG.

### Methylation analysis

**Figure 1:**
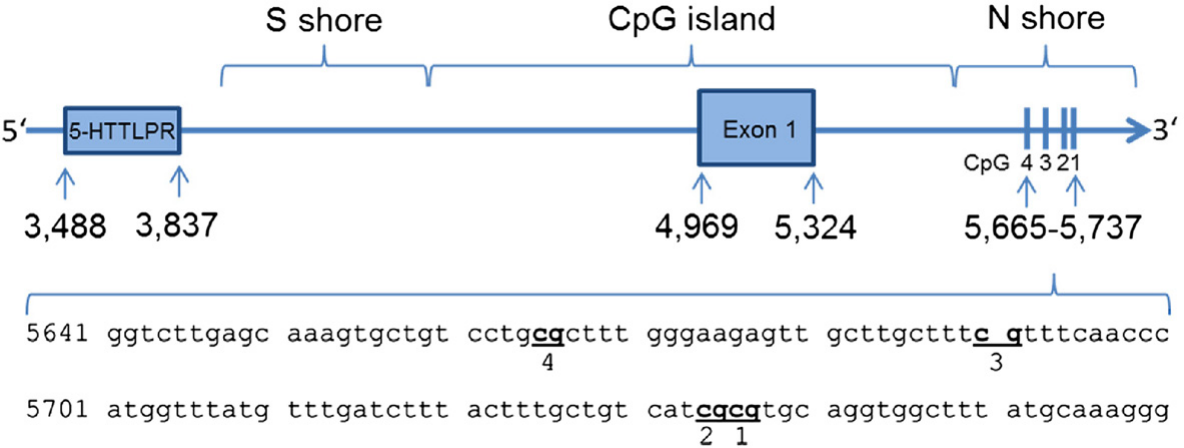
**Schematic overview and sequence of** ***SLC6A4*** **promoter associated CpG island region.** The positions refer to GeneBank accession number NG_011747.2. The numbers 1 to 4 refer to locations of the CpG sites assayed in the present study. CpG site 2 corresponds to CpG site cg22584138. (5-HTTLPR = *SLC6A4*-linked polymorphic region, S shore = South shore of CpG island, CpG island = cytosin-phosphate-guanine island, N shore = North shore of CpG island).

Ethylenediaminetetraacetic umbilical blood samples were obtained from all participants. Automated genomic DNA extraction was performed using the chemagic Magnetic Separation Module I (Chemagen Biopolymer-Technologie AG; Baesweiler; Germany). Genomic DNA samples (500 ng) from 90 individuals were bisulfit-treated using the EpiTect Bisulfite Kit (Qiagen, Hilden, Germany), and stored at −20°C prior to analysis. To obtain results comparable to those of earlier studies, the analysis focused on four of the five CpG sites described by Sugawara et al. [19]. These are located in the promoter associated CpG island north shore (Figure 1), and island shore methylation in general is likely to be related to gene expression [24]. Methylation at the analyzed region shows high variance ([25], data set 11 validation analysis module via MarmalAid-database). The CpG site two in the present study corresponds to the CpG Site cg22584138 from the Illumina HumanMethylation450 BeadChip. A fragment of 204 basepairs of the serotonin transporter gene *SLC6A4* (NG_011747.2) was amplified from 2 μl bisulfit-treated DNA using PCR (HotStar Taq DNA Polymerase, Qiagen, Hilden, Germany) and the forward primer 5′-TTTTTAGTTGTTTGGGTATTTGTGTTA-3′ and the reverse primer 5′-AAAACTTTACAACCTCTTAAAAACCC-3′. A biotin was located at the 5′ end of the reverse primer (Eurofins/MWG/Operon, Ebersberg, Germany). This was used to purify the product. Cycling parameters were as follows: 95°C for 15 minutes; 50 cycles respectively of 94°C for 30 seconds, 52°C for 30 seconds, and 72°C for 30 seconds; and a final extension of 10 minutes at 72°C. PCR products were pyrosequenced using the PyroMark Q24 system (Qiagen, Hilden, Germany) and the primers 5′-TTTTGTATAAAGTTATTTGT-3′ (CpG 1 and 2), and 5′-AATATAAATTATGGGTTGAA-3′ (CpG 3 and 4). Methylated and unmethylated EpiTect control DNA samples (Qiagen, Hilden, Germany) were used as controls for the bisulfite conversion reaction, the amplification, and the pyrosequencing reaction. Successful amplification and the specificity of the PCR products were checked on an agarose gel. Processing of the PCR amplicons for the pyrosequencing analysis was performed in accordance with the manufacturer’s protocol (Qiagen, Hilden, Germany). The percentage of methylation at each CpG site was quantified using the Pyro Q-CpG software, version 2.0.6 (Qiagen, Hilden, Germany). Methylation levels were defined in terms of the percentage of methylated DNA, as indicated by the Pyro-Q software. All measurements were made in triplicate. Values marked as unreliable by the Pyro-Q software, and values deviating by >3%-points from other replicates, were discarded. The remaining replicates were averaged.

### Statistical analysis

Two-tailed *t*-tests for independent samples (SPSS® Statistics 20) were used to compare demographic factors between the extreme groups. Nominal significance was set at α = 0.05. Data and results are expressed as means ± standard deviation (SD) or as percentages, as appropriate [22]. Quality control and association testing of methylation data were performed using the R 2.15.3 software package (http://www.r-project.org). Association tests were performed using multifactorial ANOVA models which included all variables of interest with correction for plate effects. Tests were performed for each of the four sites and for the mean of all four sites.

## Results

**Figure 2:**
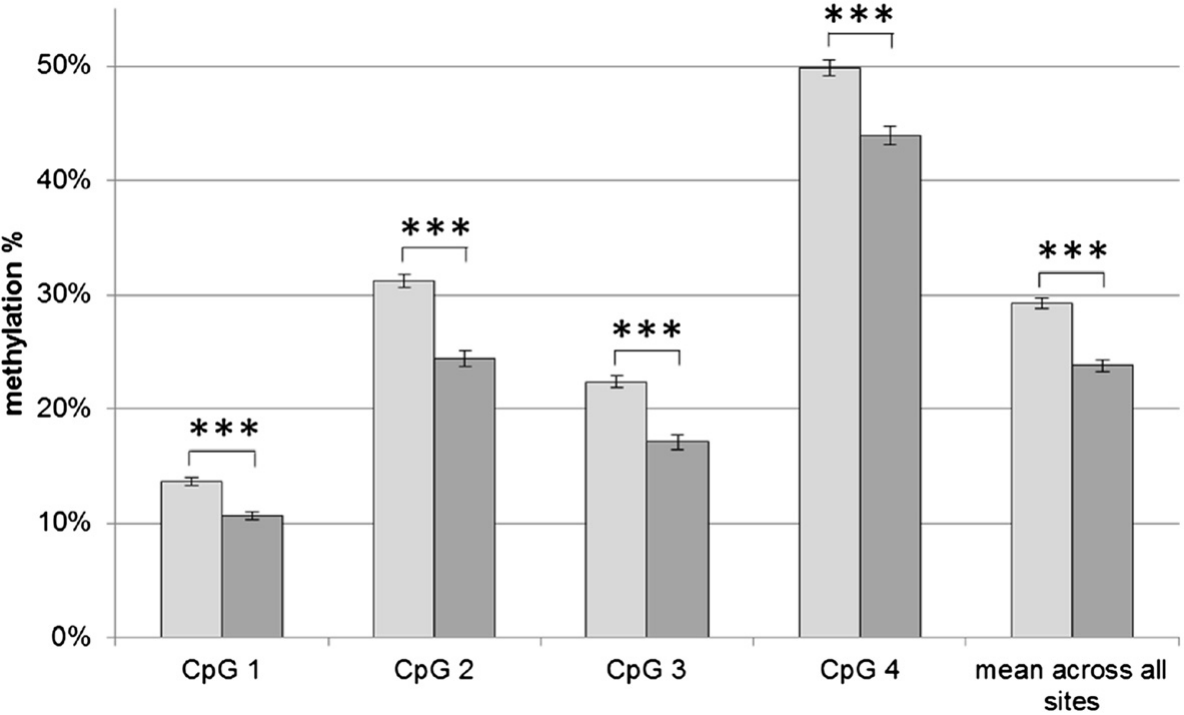
**Sex specific mean** ***SLC6A4*** **methylation.** Females shown in light gray, males in dark gray, mean in % +/- standard error of the mean. Asterisks indicate significance.

All individuals were genotyped successfully. This yielded genotype counts of 21 (LA/LA); 7 (LA/LG); 44 (LA/SA); 1 (LG/SA); and 17 (SA/SA). The genotype frequencies were LA/LA, 23.3%; LA/LG, 7.7%; LA/SA, 48.8%; LG/SA, 1.1% and SA/SA, 18.9%. The genotype distributions of 5-HTTLPR and of the combined 5-HTTLPR-rs25531 genotypes were in accordance with Hardy-Weinberg-Equilibrium (p_exact_ = 1; p_exact_ = 0.23). During quality control, 18 methylation measurements were discarded due to high variance. Multifactorial linear modeling involving simultaneous incorporation of the sex of the newborn, 5-HTTLPR genotype, and early life stress (high early life stress level: n = 24 females and n = 21 males, low early life stress level: n = 27 females and n = 18 males) revealed that only the sex of the newborn was associated with methylation level (mean methylation, p = 7.5 × 10^−10^). No association was found for genotype (mean methylation, p = 0.75) or early life stress (mean methylation, p = 0.94). On average, females (mean methylation x̄=29.3%, standard error of the mean [SEM] 0.5%), showed higher methylation levels than males (mean methylation x̄=23.8%, SEM 0.5%) (Figure 2). The mean percentage of DNA methylation across all four CpG sites was: 1) 26.5%, SEM 0.5%, for the low ELS group; and 2) 26.5%, SEM 0.5%, for the high ELS group (p = 0.94) (Table 3). In a secondary analysis, information on rs25531 was integrated. Combined 5-HTTLPR-rs25531 genotypes were grouped as high vs. low expressing according to Wendland et al. [23]. This analysis revealed no evidence for a genotype effect, or any interaction between genotype and other predictors in the model (Table 4).

**Table 3:** **P-values and r-squared values (in parentheses) obtained from multi factorial ANOVA models of the effect of sex, ELS, and 5-HTTLPR genotype on** ***SLC6A4*** **methylation**

	**CpG site**				
**Predictor in model**	**1**	**2**	**3**	**4**	**Mean across sites**
Sex	**3.9e-08 (0.302)**	**2.3e-09 (0.361)**	**1.2e-07 (0.288)**	**3.7e-08 (0.311)**	**7.5e-10 (0.371)**
ELS	0.94 (0.000)	0.67 (0.004)	0.92 (0.001)	0.96 (0.000)	0.94 (0.001)
Genotype	0.12 (0.070)	0.47 (0.028)	0.95 (0.005)	0.72 (0.027)	0.75 (0.025)
Sex x ELS	0.76 (0.001)	0.33 (0.006)	0.78 (0.003)	0.56 (0.003)	0.63 (0.002)
Sex x Genotype	0.50 (0.011)	0.61 (0.008)	0.73 (0.004)	0.51 (0.011)	0.57 (0.008)
ELS x Genotype	0.79 (0.003)	0.41 (0.013)	0.44 (0.012)	0.74 (0.005)	0.52 (0.009)

Significant p-values are shown in bold. ELS = early life stress; 5-HTTLPR genotype = *SLC6A4-*linked polymorphic region genotype.

**Table 4:** **P-values and r-squared values (in parentheses) obtained from multi factorial ANOVA models of the effect of sex, ELS, and combined 5-HTTLPR rs25531 genotype grouped according to Wendland et al. [** 23 **] on** ***SLC6A4*** **methylation**

	**CpG site**				
**Predictor in model**	**1**	**2**	**3**	**4**	**Mean across sites**
Sex	**4.5e-08 (0.300)**	**1.4e-09 (0.367)**	**9.7e-08 (0.286)**	**3.9e-08 (0.310)**	**6.3e-10 (0.371)**
ELS	0.82 (0.000)	0.68 (0.004)	0.86 (0.001)	0.87 (0.000)	0.98 (0.001)
Genotype_HL	0.11 (0.074)	0.83 (0.017)	0.66 (0.015)	0.68 (0.030)	0.76 (0.027)
Sex x ELS	0.69 (0.001)	0.43 (0.005)	0.66 (0.003)	0.56 (0.003)	0.69 (0.001)
Sex x Genotype_HL	0.67 (0.006)	0.65 (0.007)	0.67 (0.006)	0.81 (0.004)	0.71 (0.005)
ELS x Genotype_HL	0.81 (0.003)	0.58 (0.009)	0.77 (0.004)	0.84 (0.004)	0.74 (0.005)

Significant p-values are shown in bold. ELS = early life stress; Genotype_HL = 5-HTTLPR rs25531 multi marker genotype, _HL = _HighLow genotype grouping according to Wendland et al. [23].

## Discussion

The aims of the present study were to determine whether exposure to early life stress – defined as maternal stress during pregnancy – impacts *SLC6A4* methylation levels in newborns, and whether this is influenced by sex or 5-HTTLPR genotype. In the present cohort, methylation was influenced by sex, but not by early life stress or 5-HTTLPR genotype. Following the integration of the effects of rs25531, which has been reported to influence expression of *SLC6A4* [23], the results were unaltered.

Research has shown that DNA methylation can lead to persistent alterations in gene function, and that this may result in a range of psychiatric phenotypes [20]. Robust data suggest that stress has an influence on methylation, and higher *SLC6A4* methylation has been associated with reduced gene expression [26]. In individuals with the S/S genotype of 5-HTTLPR, Sugawara et al. found an inverse correlation between *SLC6A4* methylation levels and gene expression [19]. Several earlier studies had demonstrated that early life stress-induced methylation differences were influenced by genetic variation in 5-HTTLPR genotype. In the Iowa adoptee sample, early adversity was associated with differential methylation, as mediated by 5-HTTLPR genotype [9-12].

As reported with high *SLC6A4* methylation, the S allele of the 5-HTTLPR genotype leads to decreased gene expression, and has been repeatedly associated with an increased vulnerability for stress-related psychiatric disorders, such as depression and anxiety [6]. However, the present study analyzed different CpG sites, and thus cross-study comparison of results is problematic. The present study focused on the functional CpG sites reported by Sugawara et al. [19]. These were selected on the basis of their promising position in the island north shore and their reported functionality.

Sensitivity to stress changes throughout the life-span, and young adults with a history of stressful life events during the first five years of life show a particularly pronounced stress response [27]. Therefore we expected individuals exposed to stress during an even earlier period of life to show increased methlyation compared to those with no history of exposure. Interestingly, however, in the present cohort, neither early life stress nor genotype influenced methylation in utero.

However, a strong sex-effect was apparent, and recent studies have generated independent evidence for an effect of sex on *SLC6A4*. Wang et al. reported that at birth, methylation of CpG site two was higher in females than in males [28]. In a longitudinal assesssment of their cohort, the authors found persistent sex-differences two years after birth. In another study, Xu et al. found sex differences in this CpG site in the post-mortem adult brain [29]. Neither of these studies, however, had taken into account early life stress or 5-HTTLPR genotype. Sex specific DNA methylation, i.e., other than that occurring during the X-inactivation process, has been demonstrated in a number of locus-specific ([30]) and genome-wide analyses (e.g. [31]). This could represent a mechanism underlying the differential susceptibility to psychiatric disorders observed between males and females [32]. Further studies are warranted to determine both whether these methylation differences are permanent, and the contributory factors.

## Conclusions

To our knowledge, the present study is the first to demonstrate sex-determined methylation of *SLC6A4* in newborns, irrespective of early life stress status and 5-HTTLPR genotype. Given the previously reported functionality of *SLC6A4* methylation and the importance of the serotonin transporter (5-HTT) in depression, higher methylation levels during early life may contribute to the increased risk for depression observed among females.

## Additional files

Additional file 1:
**Description of psychological questionnaires and the structured diagnostic interview.**
Additional file 2: Table 1. Demographic characteristics and general medical status of mothers and infants included in the methylome analysis.
