# Supplementary material for: New-born females show higher stress- and genotype-independent methylation of SLC6A4 than males
Source: Borderline Personal Disord Emot Dysregul. 2015 Apr 15;2:8. doi: 10.1186/s40479-015-0029-6 (PMC4579500; doi:10.1186/s40479-015-0029-6)
Supplement: Additional file 1: — Description of psychological questionnaires and the structured diagnostic interview. [file 40479_2015_29_MOESM1_ESM.docx]

**Additional file 1**

**Description of psychological questionnaires and the structured diagnostic interview**

Perceived stress scale (PSS) [33]

(Cohen S, Karmarck T, Mermelstein R: **A global measure of perceived stress.** *J Health Soc Behav* 1983, **24(4):**385-396.)

The Perceived Stress Scale is a 14-item self-report questionnaire that measures the individual’s subjective evaluation of the stressfulness of situations encountered in the preceding month. The PSS comprises 14 items with a 5point Likert-type response, and measures the subjective perception of stress.

**Higher scores indicate higher perceived stress.**

Prenatal distress questionnaire (PDQ) [34]

(Yali AM, Lobel M: **Coping and distress in pregnancy: an investigation of medically high risk women.** *J Psychosom Obstet Gynaecol* 1999, **20:**39-52.)

The PDQ is a self-report questionnaire which assesses worries and concerns related to pregnancy. These include concerns about maintaining a healthy diet, weight / body image, irritating physical symptoms, emotions and relationships, and anxiety regarding labor and birth.

**Higher scores indicate higher perceived stress.**

Life experiences survey (LES) [35]

(Sarason IG, Johnson JH, Siegel JM: **Assessing the impact of life changes: development of the life experiences survey.** *J Consult Clin Psych* 1978, **46(5):**932-946.)

The purpose of the LES is to obtain a self-report of positive and negative events experienced over the previous year, as well as the perceived stress associated with those events. The LES addresses 47 life changes that are common to individuals in a wide variety of situations. Every event that occurred is coded as one “life change unit”. Positive and negative events can be totalled separately.

**The present study considered negative life events.**

Social support questionnaire (SOZ_U) [36]

(Fydrich T, Sommer G, Tydecks S, Braehler E: **Social Support Questionnaire (F-SOZU): Standardization of short form (K-14).** *Zeitschrift für Medizinische Psychologie* 2009, **18(1):**43-48.)

This questionnaire measures perceived and anticipated social support. The respondent is classified as having low, medium, or high social support based on the total score.

**Higher scores indicate higher/ better social support.**

Mini-International Neuropsychiatric Interview (M.I.N.I.) [37]

(Sheehan DV, Lecrubier Y, Sheehan KH, Amorim P, Janavs J, Weiller E, Hergueta T, Baker R, Dunbar GC: The **Mini-International Neuropsychiatric Interview (M.I.N.I.): the development and validation of a structured diagnostic psychiatric interview for DSM-IV and ICD 10. *J Clin Psychiatry* 1998, 59 Suppl 20:22-33; quiz 34-57.**

The M.I.N.I. is a short, structured diagnostic interview developed for Diagnostic and Statistical Manual of Mental Disorders, fourth Edition [DSM-IV] and International Classification of Diseases [ICD]-10 in order to explore 17 psychiatric disorders: mood disorders / suicidality / anxiety disorders / generalized anxiety disorder / panic disorder / agoraphobia / social anxiety disorder / posttraumatic stress disorder / obsessive compulsive disorder / psychotic features & disorders / substance abuse (alcohol & illicit drugs) / eating disorders / personality (antisocial) disorder.

Edinburgh postnatal depression scale (EPDS) [38]

(Cox JL, Holden JM, Sagovsky R: **Detection of postnatal depression. Development of the 10-item Edinburgh Postnatal Depression Scale.** *Br J Psychiatry* 1987, **150:**782-786.)

The EPDS was developed to assess post-partum depression but has also been used to screen for depression during the antepartum period. The EPDS relies much less on somatic questions than either the Beck depression inventory (BDI) or the Center for Epidemiologic Studies Depression Scale (CES-D). The EPDS items refer to the “past seven days” and the response format is frequency-based. The responses to the 10 items are added together to obtain a score.

**Higher scores indicate higher depressive symptoms. Scores > 10 indicate a depressive episode.**

State-trait anxiety inventory (STAI-S & STAI-T) [39]

(Spielberger CD GR, Lushene PR, Vagg PR, Jacobs GA: **Manual for the State-Trait Anxiety Inventory.** *Consulting Psychologists Press, Inc* 1983.)

The STAI is used to measure anxiety. It differentiates between a temporary or emotional *s*tate anxiety (STAI-S) and long standing personality *t*rait anxiety (STAI-T) in adults. The STAI is comprised of forty questions (20 questions each scale) with a range of four possible responses to each. The range of scores is 20-80.

**Higher scores indicate higher state or higher trait anxiety.**

Anxiety screening questionnaire (ASQ) [40]

(Wittchen HU, Boyer P: **Screening for anxiety disorders. Sensitivity and specificity of the Anxiety Screening Questionnaire (ASQ-15).** *Br J Psychiatry Suppl* 1998**:**10-17.)

The ASQ is a screening instrument for use in primary care. It contains one general question (current pain / stress / somatic complains) and six diagnostic questions (major depression / panic attacks / agoraphobia / social anxiety disorder / generalized anxiety disorder / posttraumatic stress disorder).

**Higher scores indicate higher prenatal anxiety.**
