# Supplementary material for: New-born females show higher stress- and genotype-independent methylation of SLC6A4 than males
Source: Borderline Personal Disord Emot Dysregul. 2015 Apr 15;2:8. doi: 10.1186/s40479-015-0029-6 (PMC4579500; doi:10.1186/s40479-015-0029-6)
Supplement: Additional file 2: Table 1. — Demographic characteristics and general medical status of mothers and infants included in the methylome analysis. [file 40479_2015_29_MOESM2_ESM.docx]

**Additional file 2**

Additional Table 1: **Demographic characteristics and general medical status of mothers and infants included in the methylome analysis** (all data: mean ± SD or percentage)

| **Variable** | **High prenatal ELS**  **(n = 45)** | **Low prenatal ELS**  **(n = 45)** | **p value** |
| --- | --- | --- | --- |
| Maternal Age (in years) | 29.6 ± 5.6 | 32.7 ± 3.7 | 0.02 |
| Gender of infant (female) | 53.3 % | 60 % | ns |
| Smoking during late pregnancy (3^rd^ trimester) | 17.8 % | 2.2 % | 0.01 |
| Alcohol intake during late pregnancy  (3^rd^ trimester) | 2.2 % | 2.2 % | ns |
| Primiparous | 35.6 % | 51.1 % | ns |
| Pre-Pregnancy BMI | 25.5 ± 6.9 | 24.8 ± 5.8 | 0.05 |
| Gestational diabetes | 28.9 % | 17.8 % | ns |
| Gestational age at birth (wpma) | 38.7 ± 1.7 | 39.4 ± 1.1 | ns |

wpma = weeks postmenstrual age; BMI = body mass index; SD = standard deviation; ns = not significant; % = percentage
